# A Clinico-Radiological Correlation Study in Children With Severe Community-Acquired Pneumonia: Does This Child Have Pneumonia?

**DOI:** 10.7759/cureus.106958

**Published:** 2026-04-13

**Authors:** Dnyaneshwar Potpalle, Kothakapu Rajesh Reddy, Sunita Pawar, Hari Chandana, Mummareddi Dinesh Eshwar

**Affiliations:** 1 Paediatrics, Mahavir Institute of Medical Sciences, Vikarabad, IND; 2 Paediatrics and Child Health, Mahavir Institute of Medical Sciences, Vikarabad, IND; 3 Pathology and Laboratory Medicine, Mahavir Institute of Medical Sciences, Vikarabad, IND; 4 Medicine, Dr. Patnam Mahender Reddy Institute of Medical Sciences, Hyderabad, IND; 5 General Medicine, Mahavir Institute of Medical Sciences, Vikarabad, IND

**Keywords:** bronchopneumonia, chest radiography, clinical correlation, community-acquired pneumonia, hypoxemia, pediatric pneumonia, severe pneumonia, who criteria

## Abstract

Background: Pneumonia remains a leading cause of morbidity and mortality among children under five years of age, particularly in developing countries. Although the World Health Organization (WHO) provides clinical criteria for diagnosing severe pneumonia, overlapping features with other respiratory illnesses may limit diagnostic accuracy. Radiological evaluation, especially chest radiography, plays a crucial role in confirming diagnosis, assessing severity, and identifying complications.

Materials and methods: This analytical cross-sectional study included 300 consecutively enrolled children aged 2-60 months admitted with WHO-defined severe community-acquired pneumonia. Clinical data, including demographic characteristics, presenting symptoms, and physical signs, were systematically recorded. Oxygen saturation was measured using pulse oximetry. Laboratory parameters, including complete blood counts and erythrocyte sedimentation rate, were analyzed. Chest radiographs were performed in all patients and categorized into pneumonic consolidation, interstitial pneumonia, bronchopneumonia, and associated complications. Radiographs were independently interpreted to reduce observer bias. For statistical analysis, radiological findings were categorized as a binary variable (presence or absence of any abnormality on chest X-ray), and associations between clinical parameters and radiological abnormalities were assessed using the chi-square test.

Results: The majority of patients (228, 76%) were aged 2-12 months, with a male predominance of 204 (68%). Common presenting features included cough in 295 (98.4%), hurried breathing in 300 (100%), and fever in 240 (80%), while lethargy was noted in 134 (44.8%) of cases. Clinical examination revealed tachypnea in 293 (97.6%), chest retractions in 288 (96%), crepitations in 192 (64%), wheezing in 96 (32%), and bronchial breath sounds in 84 (28%) of patients. Hypoxemia (SpO₂ <90%) was present in 192 (64%) of cases. Laboratory findings demonstrated leukocytosis in 156 (52%), neutrophilia in 168 (56%), anemia in 146 (48.8%), and elevated ESR in 185 (61.6%) of patients. Radiological abnormalities were identified in 156 (52%) of cases, with pneumonic consolidation in 138 (46%) being the most common pattern, followed by interstitial pneumonia in 102 (34%) and bronchopneumonia in 45 (15%). Complications such as pleural effusion and empyema were infrequent. A statistically significant association was observed between clinical parameters and the presence of radiological abnormalities on chest X-ray, including fever (p<0.001), hypoxemia (p<0.001), cyanosis (p=0.0097), wheezing (p=0.014), crepitations(p<0.001), and bronchial breath sounds (p<0.001).

Conclusion: Clinical features, particularly hypoxemia, bronchial breath sounds, and crepitations, show a strong association with the presence of radiological abnormalities in pediatric pneumonia. Chest radiography remains an essential tool for confirming diagnosis, characterizing disease patterns, and detecting complications. A combined clinical and radiological approach enhances diagnostic accuracy and supports effective management in children with severe community-acquired pneumonia.

## Introduction

Pneumonia is an acute lower respiratory tract infection involving the lung parenchyma and remains a major cause of morbidity and mortality among children under five years of age worldwide. Despite advances in healthcare, pneumonia continues to account for a substantial proportion of childhood deaths, particularly in developing countries. According to the World Health Organization (WHO), pneumonia contributes significantly to under-five mortality, with nearly 20% of deaths occurring in this age group, especially in low- and middle-income countries, such as India [1]. Furthermore, approximately 90% of pneumonia cases occur in resource-limited settings [2-4], where access to healthcare and appropriate treatment remains a significant challenge [5-7].

The diagnosis of pneumonia in children is primarily based on clinical criteria established by the WHO, including signs such as tachypnea, chest indrawing, hypoxemia, and general danger signs (e.g., inability to feed, persistent vomiting, convulsions, lethargy or unconsciousness, and central cyanosis) [8]. These criteria are simple and cost-effective, making them suitable for use in resource-constrained settings. However, several respiratory conditions, such as bronchiolitis and asthma, may present with similar clinical features, thereby reducing the specificity of clinical diagnosis [9]. This overlap often poses a challenge in accurately determining disease severity and appropriate management.

Radiological evaluation, particularly chest radiography, plays a crucial role as an adjunct to clinical assessment in pediatric pneumonia. Chest X-ray aids in confirming the diagnosis; identifying patterns such as consolidation, interstitial pneumonia, and bronchopneumonia; and detecting complications, including pleural effusion and empyema [10]. Previous studies have demonstrated that radiological findings correlate with disease severity and clinical outcomes, highlighting the importance of imaging in the evaluation of community-acquired pneumonia [11].

In addition to clinical and radiological evaluation, laboratory parameters, such as leukocytosis, neutrophilia, anemia, and elevated inflammatory markers, provide supportive evidence of infection and help assess disease severity. However, the correlation between clinical presentation, laboratory findings, and radiological patterns varies across populations and healthcare settings, emphasizing the need for region-specific data [12]. Furthermore, disparities in healthcare access and disease burden in low- and middle-income countries continue to influence outcomes in pediatric pneumonia [13-16].

Recent studies have also explored predictive models and epidemiological trends to improve diagnosis and risk stratification in pediatric pneumonia [17-19]. Large-scale studies have demonstrated that community-acquired pneumonia remains a leading cause of hospitalization and healthcare burden among children worldwide [20-22]. Additionally, radiological patterns have been shown to correlate with disease severity and functional impairment in respiratory conditions [23-26].

Although several studies have evaluated the clinical and radiological features of community-acquired pneumonia, there is limited data focusing on the correlation between these parameters in children with WHO-defined severe pneumonia in the Indian context. Understanding this relationship is essential for improving diagnostic accuracy, guiding appropriate investigations, and optimizing management strategies.

In this context, the present study was undertaken to evaluate the clinical and radiological characteristics of severe community-acquired pneumonia in children aged two months to five years and to determine the correlation between clinical features and radiological findings in a tertiary care hospital setting.

## Materials and methods

Study design and setting

This prospective observational analytical cross-sectional study was conducted in the Department of Pediatrics at Mahavir Institute of Medical Sciences, Vikarabad, India, over a period of one year, from April 2025 to March 2026. The study protocol was approved by the Institutional Ethics Committee (IEC) of Mahavir Institute of Medical Sciences, Vikarabad (IEC/Proposal/2025/149). Written informed consent was obtained from the parents or legal guardians of all participants prior to enrollment.

Study population

A total of 300 consecutive children aged 2-60 months admitted with WHO-defined severe community-acquired pneumonia were included in the study. The diagnosis of severe pneumonia was made according to the World Health Organization Integrated Management of Childhood Illness (IMCI) guidelines [18].

Sample size calculation

The sample size was calculated using the standard formula for estimating proportions:

$n = (Z&sup2; &times; p &times; q) / d&sup2;$

Here, n is the required sample size, Z is the standard normal variate corresponding to a 95% confidence level (Z = 1.96), p is the estimated prevalence of radiological abnormalities in severe pneumonia based on previous epidemiological studies [23], q = 1 − p, and d is the margin of error (set at 5%). Based on these assumptions, the minimum required sample size was calculated to be approximately 273 patients. To enhance the statistical power and reliability of the study, a total of 300 patients were included.

Inclusion criteria

Children aged 2-60 months presenting with cough or difficulty in breathing, along with signs of severe pneumonia, such as tachypnea, lower chest wall indrawing, central cyanosis, inability to feed, altered sensorium, or oxygen saturation (SpO₂) less than 90% on room air, were included in the study.

Exclusion criteria

Children with congenital lung anomalies, chronic lung diseases such as bronchopulmonary dysplasia or cystic fibrosis, known immunodeficiency disorders, hospital-acquired pneumonia, or incomplete clinical records were excluded from the study.

Data collection and clinical evaluation

All enrolled patients underwent detailed clinical evaluation using a structured proforma based on WHO IMCI criteria, which included all the abovementioned clinical parameters. Demographic details, including age and sex, were recorded. Clinical features, such as fever, cough, tachypnea, chest indrawing, wheezing, cyanosis, refusal of feeds, and altered sensorium, were documented. Vital parameters, including respiratory rate, temperature, and SpO₂ measured by pulse oximetry, were recorded. Respiratory system examination was performed to assess crepitations, wheezing, bronchial breath sounds, decreased breath sounds, and grunting.

Laboratory investigations

Laboratory investigations, including hemoglobin, total leukocyte count, differential leukocyte count, and erythrocyte sedimentation rate, were performed in all patients. Microbiological investigations, such as blood culture, sputum examination, and pleural fluid analysis, were carried out wherever clinically indicated using standard laboratory methods.

Radiological evaluation

Chest radiography was performed in all patients at admission. Radiographs were independently interpreted by two experienced radiologists to minimize interobserver variability. Radiological findings were categorized into pneumonic consolidation, interstitial pneumonia, bronchopneumonia, and associated complications such as pleural effusion, empyema, and lung collapse. Radiological abnormality was defined as the presence of any abnormal chest X-ray finding. For statistical analysis, radiological findings were categorized as a binary variable (presence or absence of radiological abnormality on chest X-ray).

Treatment and follow-up

All patients were managed as per standard hospital treatment protocols, which included parenteral antibiotics, oxygen supplementation, intravenous fluids, and supportive care. The study did not involve any intervention or alteration in treatment, and all patients were treated according to existing clinical guidelines. Clinical progress was monitored during hospitalization, including improvement in symptoms, normalization of SpO₂, and resolution of respiratory distress.

Statistical analysis

Continuous data were expressed as mean ± standard deviation, and categorical data were presented as frequencies and percentages. The chi-square test was used to determine the association between clinical parameters and the presence of radiological abnormalities on chest X-ray. A p-value of less than 0.05 was considered statistically significant. Statistical analysis was performed using Statistical Product and Service Solutions (SPSS, version 25.0; IBM SPSS Statistics for Windows, Armonk, NY).

## Results

A total of 300 children with severe community-acquired pneumonia were included in the study. The majority of patients were in the 2-12-month age group, accounting for 76% (228/300) of cases, while 24% (72/300) were aged between 13 and 59 months. This indicates a higher vulnerability of infants to severe pneumonia. There was a clear male predominance, with males comprising 68% (204/300) and females 32% (96/300), suggesting a possible gender-related susceptibility, as shown in Table 1.

**Table 1:** Age and gender distribution of children with severe community-acquired pneumonia (n=300)

Age (months)	Female, n (%)	Male, n (%)	Total, n (%)
2-12	78 (34%)	150 (66%)	228 (76%)
13-59	18 (26%)	54 (74%)	72 (24%)
Total	96 (32%)	204 (68%)	300 (100%)

The demographic profile was followed by an analysis of presenting clinical features. The most common presenting symptoms were hurried breathing (100%), cough (98.4%), and fever (80%), highlighting the typical clinical presentation of severe pneumonia. Refusal of feeds was observed in 40% (120/300) of patients, while lethargy was noted in 44.8% (134/300), indicating systemic involvement. Cyanosis (4.8%), convulsions (3.2%), and stridor (0.8%) were relatively less frequent but represent severe disease manifestations (Table 2).

**Table 2:** Distribution of presenting clinical symptoms among children with severe community-acquired pneumonia (n=300)

Symptom	Number	Percentage (%)
Hurried breathing	300	100
Cough	295	98.4
Fever	240	80
Refusal of feeds	120	40
Lethargy	134	44.8
Cyanosis	14	4.8
Convulsions	10	3.2
Stridor	2	0.8

On clinical examination, tachypnea (97.6%) and chest retractions (96%) were the most consistent findings, reinforcing their importance as key diagnostic criteria for severe pneumonia. Crepitations were noted in 64% of cases, indicating alveolar involvement, while wheezing (32%) suggests airway involvement or mixed pathology. Bronchial breath sounds were present in 28% of patients, indicating consolidation. Grunting (16%) and decreased breath sounds (8%) were less frequent but signify severe respiratory compromise (Table 3). Hypoxemia (SpO₂ <90%) was observed in 64% (192/300) of patients, emphasizing its role as a critical marker of disease severity.

**Table 3:** Distribution of clinical signs among children with severe community-acquired pneumonia (n=300)

Sign	Number	Percentage (%)
Tachypnea	293	97.6
Chest indrawing	288	96
Crepitations	192	64
Wheeze	96	32
Bronchial breath sounds	84	28
Grunting	48	16
Decreased breath sounds	24	8

Laboratory investigations revealed leukocytosis in 52% (156/300) of patients and neutrophilia in 56% (168/300), suggesting a predominant bacterial etiology. Anemia was present in 48.8% (146/300), which may contribute to increased disease severity, while elevated erythrocyte sedimentation rate (61.6%) reflects ongoing inflammation. Lymphocytosis was observed in 33.6% (101/300) of cases (Table 4).

**Table 4:** Hematological parameters among children with severe community-acquired pneumonia (n=300) Hb: Hemoglobin (g/dL); anemia defined as Hb <11 g/dL; leukocytosis defined as total leukocyte count >11,000 cells/mm³; neutrophilia defined as neutrophils >60%; lymphocytosis defined as lymphocytes >40%; ESR: erythrocyte sedimentation rate (mm/hr), elevated ESR defined as >20 mm/hr

Parameter	Number	Percentage (%)
Anemia	146	48.8
Leukocytosis	156	52
Neutrophilia	168	56
Lymphocytosis	101	33.6
Elevated ESR	185	61.6

Radiological evaluation demonstrated abnormalities in 52% (156/300) of patients. Pneumonic consolidation was the most common pattern (46%), indicating bacterial pneumonia as the predominant form. Interstitial pneumonia (34%) suggests viral or atypical etiology, while bronchopneumonia (15%) reflects patchy involvement. Complications were relatively uncommon, with pleural effusion (2%) and empyema (1%), indicating that most cases were identified before advanced complications developed (Table 5).

**Table 5:** Chest radiographic findings in children with severe community-acquired pneumonia (n=300)

Finding	Number	Percentage (%)
Consolidation	138	46
Interstitial pneumonia	102	34
Bronchopneumonia	45	15
Pleural effusion	6	2
Empyema	3	1
Collapse	0	0

Statistical analysis revealed significant associations between clinical parameters and the presence of radiological abnormalities on chest X-ray (Table 6). Fever (χ²=19.28, p<0.001), hypoxemia (χ²=93.48, p<0.001), cyanosis (χ²=6.69, p=0.0097), crepitations (χ²=15.38, p<0.001), wheezing (χ²=6.04, p=0.014), and bronchial breath sounds (χ²=45.89, p<0.001) showed statistically significant associations.

**Table 6:** Association between clinical parameters and presence of radiological abnormalities on chest X-ray in children with severe community-acquired pneumonia (n=300) Radiological abnormality was defined as the presence of any abnormal chest X-ray finding, including consolidation, bronchopneumonia, interstitial pneumonia, or complications such as pleural effusion and empyema. Statistical analysis was performed using the chi-square test. A p-value <0.05 was considered statistically significant. *Statistically significant (p < 0.05)

Clinical Parameter	Status	Radiological Abnormality (n = 156)	No Radiological Abnormality (n = 144)	χ² value	p-value
Fever	Present	140	100	19.28	<0.001*
	Absent	16	44		
Cough	Present	154	141	0.29	0.588
	Absent	2	3		
Poor Feeding	Present	60	60	0.32	0.571
	Absent	96	84		
Lethargy	Present	70	64	0.005	0.941
	Absent	86	80		
Tachypnea	Present	152	141	0.08	0.783
	Absent	4	3		
Hypoxemia	Present	140	52	93.48	<0.001*
	Absent	16	92		
Cyanosis	Present	12	2	6.69	0.0097*
	Absent	144	142		
Vomiting	Present	20	20	0.07	0.786
	Absent	136	124		
Crepitations	Present	120	80	15.38	<0.001*
	Absent	36	64		
Wheezing	Present	40	56	6.04	0.014*
	Absent	116	88		
Chest Retractions	Present	150	138	0.02	0.887
	Absent	6	6		
Bronchial Breath Sounds	Present	70	14	45.89	<0.001*
	Absent	86	130		
Decreased Breath Sounds	Present	16	8	2.25	0.134
	Absent	140	136		

In contrast, clinical parameters, such as cough (p=0.588), poor feeding (p=0.571), lethargy (p=0.941), tachypnea (p=0.783), vomiting (p=0.786), chest retractions (p=0.887), and decreased breath sounds (p=0.134), did not show statistically significant associations.

Overall, clinical features, such as hypoxemia and bronchial breath sounds, demonstrated strong associations with the presence of radiological abnormalities on chest X-ray, while cyanosis, wheezing, and crepitations also showed statistically significant associations.

## Discussion

The management of pediatric pneumonia is guided by established global recommendations that emphasize severity-based classification and timely intervention. According to the WHO guidelines, community-acquired pneumonia is categorized based on clinical features, with severe pneumonia characterized by tachypnea and chest indrawing, and very severe pneumonia, including additional danger signs, such as hypoxemia, cyanosis, and altered sensorium. Despite the widespread use of clinical criteria, variability in presentation and overlap with other respiratory conditions necessitate adjunctive investigations, particularly radiological imaging, to improve diagnostic accuracy.

In the present study, the majority of patients were in the 2-12-month age group, indicating increased susceptibility of infants to severe lower respiratory tract infections. Similar age distribution has been reported by Tuta-Quintero et al., who demonstrated higher disease burden in younger age groups [11], and by Zar et al., who highlighted increased vulnerability in early childhood due to immature immunity and airway factors [13]. A clear male predominance was also observed, which is in agreement with findings reported by Zar et al. [13] and Dean et al. [14], suggesting a higher incidence of respiratory infections among male children. This may be due to inherent biological differences in immune response, as well as sociocultural factors influencing healthcare access.

The clinical profile observed in our study, with cough, fever, and hurried breathing being the most common presenting symptoms, is consistent with WHO diagnostic criteria and findings reported by Martin et al. [17] and WHO guidelines [18]. Tachypnea and chest retractions were the most consistent clinical signs, reaffirming their importance as key indicators of severe pneumonia. However, despite their high prevalence, these parameters did not show a statistically significant association with the presence of radiological abnormalities on chest X-ray. This finding aligns with observations by Lozano et al. [27], who demonstrated that commonly used clinical signs may lack specificity for radiological pneumonia.

Hypoxemia was observed in a significant proportion of patients and demonstrated a strong association with the presence of radiological abnormalities on chest X-ray. Similar findings have been reported by Walker et al. and Duke, who identified hypoxemia as a key indicator of severe disease and poor oxygenation due to alveolar involvement [23,24]. The strong association between hypoxemia and radiological abnormalities in our study highlights its utility as a predictor of underlying parenchymal involvement.

Laboratory findings revealed leukocytosis and neutrophilia in a majority of patients, suggesting a predominantly bacterial etiology in severe cases. Elevated erythrocyte sedimentation rate and the presence of anemia further reflect systemic inflammation and underlying nutritional compromise. Comparable hematological patterns have been reported by Shah et al. and Jain et al., who demonstrated similar inflammatory profiles in pediatric pneumonia [20,22]. The high prevalence of anemia observed in this study may also contribute to tissue hypoxia and increased disease severity.

Radiological abnormalities were identified in approximately half of the study population. Pneumonic consolidation was the most common radiological pattern observed, supporting the predominance of bacterial pneumonia in severe cases. Similar radiological distributions have been reported by Begum et al., who identified consolidation as the most frequent finding in severe pneumonia [10], and by Tuta-Quintero et al., who also noted variability in radiographic patterns depending on disease severity [11]. Interstitial pneumonia and bronchopneumonia in our study further indicate a spectrum of etiologies, including viral and mixed infections.

In the present study, radiological findings were analyzed as a binary outcome, defined as the presence or absence of abnormalities on chest X-ray. This approach was adopted to evaluate the association between clinical parameters and overall radiological involvement rather than individual radiological patterns. Hypoxemia, bronchial breath sounds, and crepitations demonstrated strong associations with radiological abnormalities, while cyanosis and wheezing showed moderate but statistically significant associations. In particular, bronchial breath sounds demonstrated a strong association, consistent with the classical clinical-radiological correlation observed in consolidation. Wheezing may reflect airway involvement or mixed pathology, explaining its association.

Interestingly, certain clinical parameters, such as cough, tachypnea, chest retractions, lethargy, and vomiting, did not show a statistically significant association with radiological abnormalities. Similar findings have been reported by Lozano et al. [27], reinforcing the limitation of relying solely on clinical examination to predict radiological involvement.

The relatively low incidence of complications such as pleural effusion and empyema in our study may be attributed to early presentation and timely management. Early intervention likely prevented progression to advanced disease stages. Although detailed microbiological profiling was not performed in all cases, the predominance of consolidation suggests a likely bacterial etiology, consistent with findings reported by Begum et al. and Mehta et al. [10,25].

The present study addresses an important gap in existing literature by evaluating the association between clinical parameters and radiological abnormalities in children with severe pneumonia. While previous studies have often assessed these aspects independently, our study provides an integrated perspective on clinical-radiological correlation.

The findings of this study have important clinical implications, particularly in resource-limited settings. While the WHO clinical criteria remain essential for early diagnosis, the addition of chest radiography enhances diagnostic accuracy and facilitates early detection of complications. Integration of clinical and radiological assessment can therefore improve patient management.

However, this study has certain limitations. Being a single-center study, the findings may not be generalizable. The observational design may introduce bias. Additionally, etiological confirmation using advanced microbiological or molecular techniques was not performed, limiting differentiation between bacterial and viral causes. Advanced inflammatory markers, such as C-reactive protein (CRP) and procalcitonin (PCT), were not included due to resource constraints. Molecular diagnostic methods, such as polymerase chain reaction (PCR), were also not utilized. Radiological findings were analyzed as a binary variable, and individual radiological patterns were not assessed separately due to a lack of detailed cross-tabulated data. The absence of long-term follow-up also limits outcome assessment.

Despite these limitations, the present study provides valuable insights into the clinical and radiological spectrum of severe community-acquired pneumonia in children and highlights the importance of integrated diagnostic approaches.

## Conclusions

Pneumonia continues to be a major cause of morbidity and mortality among children under five years of age, particularly in developing countries with limited healthcare resources. Early and accurate diagnosis remains crucial for effective management and prevention of complications. While clinical assessment based on the WHO criteria forms the cornerstone of diagnosis, it may not reliably predict the presence or extent of pulmonary involvement. In the present study, significant associations were observed between clinical parameters and the presence of radiological abnormalities on chest X-ray in children with severe community-acquired pneumonia. Clinical features, such as hypoxemia and bronchial breath sounds, were found to be strong predictors of radiological abnormalities, while cyanosis, wheezing, and crepitations also demonstrated statistically significant associations. In contrast, commonly used signs, such as tachypnea and chest retractions, although sensitive, were not specific for radiological involvement. Pneumonic consolidation emerged as the most common radiological pattern, suggesting a predominance of bacterial etiology in severe cases.

These findings highlight the importance of integrating clinical evaluation with radiological assessment to improve diagnostic accuracy, facilitate early identification of complications, and guide appropriate management strategies. Such an approach is particularly valuable in resource-constrained settings, where timely decision-making can significantly impact outcomes. Further multicentric studies with larger sample sizes and incorporation of etiological and microbiological analysis are recommended to enhance understanding of disease patterns and optimize management protocols in pediatric pneumonia.
