# Correction: A Clinico-Radiological Correlation Study in Children With Severe Community-Acquired Pneumonia: Does This Child Have Pneumonia?

**DOI:** 10.7759/cureus.c455

**Published:** 2026-06-26

**Authors:** Dnyaneshwar Potpalle, Kothakapu Rajesh Reddy, Sunita Pawar, Hari Chandana, Mummareddi Dinesh Eshwar

**Affiliations:** 1 Paediatrics, Mahavir Institute of Medical Sciences, Vikarabad, IND; 2 Paediatrics and Child Health, Mahavir Institute of Medical Sciences, Vikarabad, IND; 3 Pathology and Laboratory Medicine, Mahavir Institute of Medical Sciences, Vikarabad, IND; 4 Medicine, Dr. Patnam Mahender Reddy Institute of Medical Sciences, Hyderabad, IND; 5 General Medicine, Mahavir Institute of Medical Sciences, Vikarabad, IND

This article has been corrected to fix the following errors:

- Replace all seven instances of the word “palpitations” with “crepitations”, including in Table 6.
- The study duration has been corrected from "April 2024–March 2025" to "April 2025–March 2026" in the Materials & Methods section.

